# Therapeutic hypothermia with rapid thin liquid convection is safe and feasible in acute ischemic stroke patients: the SISCO pilot study

**DOI:** 10.3389/fneur.2025.1611794

**Published:** 2025-11-07

**Authors:** Justin A. Salerian, Robert B. Schock, Clemens M. Schirmer, Souvik Sen, Sheryl Martin-Schild, Oded Goren, Douglas F. Kupas, Robert J. Freedman, Aimee Aysenne

**Affiliations:** 1 School of Medicine, Tulane University, New Orleans, LA, United States; 2 Life Recovery Systems, Kinnelon, NJ, United States; 3 Department of Neurosurgery and Neuroscience Institute, Geisinger Health System and Geisinger College of Health Sciences, Danville, PA, United States; 4 Department of Neurology, University of South Carolina, Columbia, SC, United States; 5 Dr. Brain, Inc., New Orleans, LA, United States; 6 Department of Emergency Medicine, Geisinger Health System, New Orleans, LA, United States

**Keywords:** hypothermia, ischemic stroke, cytoprotection, pneumonia, stroke, therapeutics

## Abstract

**Background:**

Laboratory studies have shown that rapid therapeutic hypothermia (TH) of <34 °C can reduce stroke infarct volume by over 50%. The EuroHyp-1 and ICTUS 2/3 trials found no benefits in the slow cooling of ischemic stroke patients, while extensive shivering was observed. More powerful cooling methods are required to improve outcomes.

**Methods:**

In a feasibility study approved to include up to 30 patients, the ThermoSuit^®^ System (TSS) was used to cool sedated ischemic stroke patients to 32–34 °C. Patients were cooled after reperfusion, and TH was maintained for 24 h. Cooling speeds, adverse events, and neurological outcomes [including Modified Rankin Score (mRS) at 90 days] were documented.

**Results:**

The trial was terminated after enrolling 14 subjects from 3 sites after meeting the study feasibility criteria. Ten subjects qualified for outcomes analysis. All cooled patients reached the 34 °C target with a median time of 40 min. Patients cooled under this protocol showed no increased harm and trends for improved neurological outcomes compared to previously published studies. A pneumonia rate of 23% (3/13) was comparable to prior studies in which stroke patients were cooled. Brief shivering occurred in most patients but was limited to 5.4% of the time while in the hypothermic temperature range. Patients cooled per the SISCO protocol had a rate of acceptable outcomes (mRS ≤ 3) of 90%. In intention-to-treat analysis, 82% of patients had acceptable outcomes.

**Discussion:**

The TSS is a feasible tool to achieve TH in ischemic stroke patients. This non-invasive cooling technique allows swift cooling of patients to ≤34 °C with little shivering and no apparent safety issues. Further studies are warranted to prove this rapid cooling method is cytoprotective in stroke patients, as evidenced by improved odds of functional independence.

**Clinical trial registration:**

## 1 Introduction

Acute ischemic stroke remains a leading cause of morbidity and mortality worldwide despite improved reperfusion treatments. Neuroprotection through therapeutic hypothermia (TH) remains a promising approach to managing this disease. TH is a potent neuroprotective treatment in animal stroke models because thermodynamic principles slow or block deleterious metabolic processes and target multiple signaling processes in the ischemic cascade (1–3). Data suggest that cooling profoundly impacts cellular metabolism and favorably alters many biochemical processes associated with post-ischemic neuronal injuries (4).

In animal models, TH at 3 to 4 C° below normothermia reduced infarct volume by up to 59% when initiated immediately, 49% when initiated 2 h post-reperfusion, and 28% when initiated 4 h post-reperfusion; all cytoprotective benefits were lost at 6 h post-reperfusion (5). TH of less than 34 °C reduces brain infarction size. The optimal temperature range for infarct size reduction is 34 to 31 °C applied early post-injury (4). In humans, TH in the range of 32–34 °C is neuroprotective in neonates with hypoxic–ischemic encephalopathy and possibly beneficial in comatose adults shortly after resuscitation from cardiac arrest (6, 7). Rapid TH has been more challenging to achieve in stroke patients (8).

Randomized controlled trials failed to show the benefit of mild TH in awake patients without mechanical thrombectomy. The ICTuS 2 trial was planned as a 1,600-patient study to use cooling catheters and cold infusions to cool ischemic stroke patients to a target of 33 °C (9). The trial was halted prematurely. Of the 120 patients who were enrolled, the 63 cooled patients showed trends for worsened neurological recovery, higher mortality, and a higher incidence of pneumonia compared to the 57 who were not cooled. Most patients did not reach the target temperature until at least 5 h after the alteplase bolus, with some requiring over 16 h of cooling to achieve the target temperature. The authors suggested that faster methods might be beneficial.

The EuroHYP-1 study was designed to cool ischemic stroke patients to a target temperature of 34 to 35 °C (10). In most cases, temperatures in the 34–35 °C range could not be maintained due to shivering. The study was stopped after 98 of the planned 1,500 patients were enrolled, with no observable benefit of the cooling treatment. More recently, the Intrepid study failed to demonstrate benefit for stroke patients from euthermia (T < 38 °C) using the Bard Artic Sun device, and yet still experienced high shivering rates: 85.5% in the fever prevention group and 24.3% in the control group (11).

The SISCO Study (Helping Stroke Patients with ThermoSuit Cooling) investigates a faster method of reaching the target temperature. The ThermoSuit^®^ System (TSS) employs surface cooling via direct contact cold liquid convection to rapidly achieve target temperatures. The U.S. Food and Drug Administration (FDA) has cleared the TSS for patient temperature reduction where clinically indicated, e.g., for hyperthermic patients. Research in post-cardiac arrest patients has reported typical cooling times of 30–40 min to reach ≤34 °C. This is considerably faster than the cooling times reported in other TH studies (12). The investigators of the SISCO study hypothesized that this faster cooling would improve the feasibility of reaching temperatures below 34 °C within the ideal time window identified in preclinical stroke studies. The SISCO study evaluates the safety and feasibility of the TSS to induce therapeutic hypothermia in acute stroke patients rapidly.

## 2 Methods

**Figure 1:**
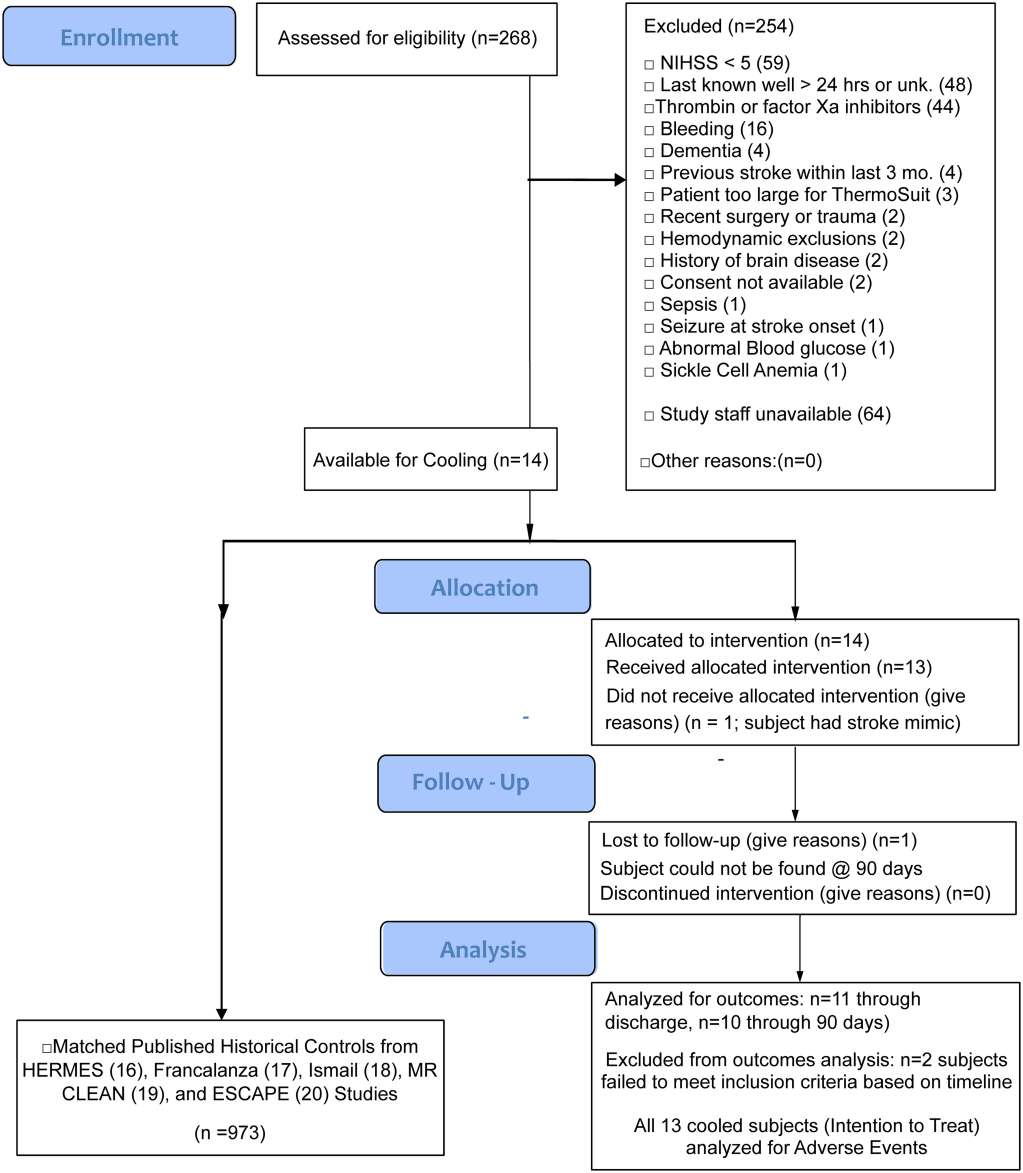
CONSORT flow chart summarizing sequence of SISCO study.

The SISCO study (Protocol No. LRS-01-13-01) is designed to assess the safety and feasibility of TSS in inducing rapid TH in up to 30 acute ischemic stroke patients. The trial was registered on ClinicalTrials.gov, https://clinicaltrials.gov/study/NCT02453373. The study size of 30 was mutually agreed upon by FDA and LRS (Life Recovery Systems, the manufacturer of the TSS). This is a typical pilot study size used for new interventions. Published recommendations for pilot study sample sizes typically range from 12 to 30 or more per group (13). A more recent publication has described additional methods for estimating sample sizes of both pilot and definitive studies (14). Life Recovery Systems and the independent Comprehensive Research Associates Data Safety Monitoring Board monitored study safety. This non-randomized feasibility protocol study was conducted according to applicable CONSORT guidelines for pilot and feasibility trials (15). A CONSORT flow chart summarizing SISCO study participant enrollments and analysis is provided in Figure 1. Any conclusive statistical analysis of the outcomes was not expected, given the small sample size. Still, they were expected to suffice to decide whether a randomized, prospectively controlled trial should be pursued.

Patients presenting to the hospital with clinical signs and symptoms of acute ischemic stroke underwent standard clinical initial evaluation. They were then screened for study eligibility using the inclusion and exclusion criteria listed in Table 1. If all inclusion criteria and no exclusion criteria were present, informed consent was sought from the patient or a legally authorized representative.

**Table 1:** Inclusion/exclusion criteria for the SISCO study.

Inclusion criteria	Exclusion criteria
1. Age ≥ 18 years 2. Ischemic stroke with NIHSS > 5 3. Treatment initiated within 8 h from known time of onset or, for eligible patients under the current AHA Guidelines, within the extended time window for mechanical thrombectomy of up to 24 h. 4. Patient dimension criteria: Height: 140–190 cm (58–75 in); Width: ≤ 66 cm (26 in) (elbow to elbow). 5. Cooling is initiated as soon as possible after reperfusion therapy.	1. Sepsis (bacteremia and clinical syndrome within 72 h). 2. Known preexisting coagulopathy, (INR > 1.3, PTT > 1.5 x control), active bleeding of unknown cause, immune compromised state, thrombocytopenia (platelet count < 160,000/mm), and history of cold agglutinin Disease. 3. Hemodynamically significant cardiac dysrhythmias (e.g., QTc interval >450 msec), bradycardia (heart rate less than 50), Mobitz Type II second degree AV block (or higher AV block), and severe ventricular dysrhythmias (sustained VT or VF) which cause significant hypotension (SBP ≤ 120 mmHg) requiring more than two pressor medications. 4. Preexistent illness with life expectancy, <6 months 5. Pregnancy 6. Rapidly improving symptoms. 7. Melena, or gross hematuria 8. Sickle cell disease 9. Temperature < 35 °C on admission to ER 10. Recent (< 1 week) incisions. 11. Any intracerebral hemorrhage 12. A history of a brain vascular lesion (e.g., aneurism or arteriovenous malformation). 13. A history of brain disease or damage (e.g., neoplasm or dementia) 14. Patients receiving IV tPA > 3 h from stroke onset

Patients were treated for acute ischemic stroke consistent with consensus guidelines, including the use of intravenous thrombolysis (IVT) and/or endovascular thrombectomy (EVT) before enrollment in this trial. The initial protocol excluded patients receiving EVT, but this exclusion was removed after the first four subjects to increase the rate of enrollment. To achieve TH, the TSS device pumps cold water (0–8 °C) over and under the patient within a flexible enclosure. Moderate sedation, including propofol, dexmedetomidine, midazolam, and/or fentanyl, was used to ensure patient tolerance and comfort. All reasonable efforts were made to avoid hypotension. After any indicated reperfusion therapy, hypothermia was initiated as soon as possible after informed consent was obtained. Safety monitoring included vital signs, end-tidal CO_2_, and shivering.

Each patient was positioned in the TSS enclosure in the supine position. Core temperature was monitored through a nasopharyngeal or esophageal temperature probe. Cooling was initiated by circulating ice-cold water (0–8 °C) through the TSS enclosure at 13 L/min.

The TSS console purged the fluid from the TSS enclosure when the patient’s core temperature reached approximately 34 °C, after which the patient was removed from the device. The purge temperature was 34.5 °C for the first three cooled patients, 34.0 °C for the next three, and 33.5 °C for the final seven. The progressive reductions in purge temperature were made to ensure that the patient temperature dropped quickly below 34 °C and remained below that temperature post-induction. Sedatives and analgesics continued to be administered for patient comfort as needed. Temperature control was maintained to a target of 32–34 °C using a commercially available surface cooling/warming device (e.g., Gentherm Blanketrol^®^, Bard’s Artic Sun™, or 3 T Medical’s Altrix^®^). After 24 h of hypothermia, patients were rewarmed at a rate of approximately 0.3 °C per hour to 36.5 °C.

The primary neurologic outcome in this study was a modified Rankin Scale (mRS) at 90 ± 10 days post-stroke. For this study, outcomes were based on an mRS score of mRS ≤ 2, defined as a “good outcome,” mRS ≤ 3 as an “acceptable outcome,” and mRS ≥ 4 as an “unacceptable outcome.” Secondary outcomes in the study included NIHSS and Euro-QOL quality of life scores (EQ-5D) at 90 days (16). Neurological outcomes and rates of adverse events observed for the cooled study patients were compared to data from previously published studies of normothermic but otherwise comparable stroke patients. Studies selected for comparison enrolled ischemic stroke patients with similar average ages (±2 years) and NIHSS scores upon admission (±2 points) to those of the SISCO study. Adverse events were documented if they occurred. Premature study termination was to be allowed should the cooling treatment be associated with adverse events.

### 2.1 Potential sources of bias

The examiners and reviewers were not blinded to the treatment. The neurological outcomes measured for SISCO patients are consistent with standardized quantitative neurological testing. The comparative studies may have introduced bias as the patients in those studies did not have all the same inclusion/exclusion criteria of the SISCO study; for example, the SISCO study excluded patients with blood pressures on admission below 120 mmHg, as well as those showing rapidly improving symptoms – the comparative studies did not exclude such patients. Additionally, the evolution of clinical practice has allowed for more advanced stroke treatment options, including expanded thrombectomy timelines, to be available for SISCO patients. For these reasons, a prospective randomized trial of sufficient size will be needed to draw significant conclusions about the effectiveness of the TSS rapid cooling treatment in ischemic stroke patients. The SISCO study was designed to determine whether such a trial should be pursued and to estimate the enrollment requirements for such a study if conducted.

### 2.2 Statistical methodology

Physiological variables for all cooled patients—including clinical exam, temperature, mean arterial blood pressure, and pulse rate were recorded, and means ± standard deviations were plotted. These values were calculated using Microsoft Excel software (Version 2405) throughout the therapeutic hypothermia period to capture trends in vital parameters. This descriptive approach provided an overview of the physiological response patterns to the intervention.

Fisher’s Exact Test was used to examine adverse events, such as progressive stroke incidence, pneumonia, and unacceptable outcomes to evaluate differences in event rates between the study group and patients from a comparative trial.

The RR (relative risk) for a given outcome was calculated by dividing the percentage of SISCO study patients achieving that outcome by the percentage of patients from the comparative study (or study group) achieving the same outcome. mRS and mortality of cooled patients were compared with those of a group of comparable non-cooled studies. Weighted averaging of that group was used to match the percentages of ischemic etiologies with those of the SISCO patients. The calculation of a weighted average of outcomes was performed as follows (this example assumes the outcome of mRS ≤ 3):


$$\begin{array}{l} \text{Weighted average percentage of}\;\mathrm{mRS}\leq 3\;\text{of}\;\\ \text{three comparable studies}=(A\times D)+(B\times E)+(C\times F) \end{array}$$

where, A = % of SISCO patients with ischemic etiology 1; B = % of SISCO patients with ischemic etiology 2; C = % of SISCO patients with ischemic etiology 3; D = % of patients from first comparable study (all having ischemic etiology 1) achieving mRS ≤ 3; E = % of patients from second comparable study (all having ischemic etiology 2) achieving mRS ≤ 3; F = % of patients from third comparable study (all having ischemic etiology 3) achieving mRS ≤ 3.

A sample size estimate for a possible future pivotal study was calculated based on comparison of the percentage of acceptable outcomes for intention to treat SISCO patients vs. the percentage of acceptable outcomes from comparable studies. The calculation was made using a test for two proportions, assuming 90% power at *α* = 0.05.

Only median and interquartile range (IQR) data were available for comparative trial data on the subject’s NIHSS scores, patient age, and EQ-5D scores at 90 days. The corresponding SISCO study data were also summarized using medians and IQRs, enabling meaningful comparisons. Statistical analyses were performed using the Mann–Whitney U Test. All statistical analyses were conducted using Minitab^®^ software (Version 22.1).

The SISCO study involves a small patient sample and more restrictive inclusion criteria than the comparative clinical trials, so these analyses are not intended to establish the efficacy of TSS treatment. The results were used to determine whether a true efficacy study should be conducted in the future.

### 2.3 Study success criteria

The study was to be judged successful if the following criteria were met:

1. *Feasibility*: At least 50% of patients cooled to the target temperature range of 32–34 °C in 1 h from the start of cooling or faster, and 90% of patients cooled to the target within 2 h.
2. *Safety*: Rates of adverse events were not significantly higher than those in comparative published trials of stroke patients with similar inclusion criteria.
3. *Neurological Recoveries Compared to Comparative Clinical Trials*: mRS and NIHSS scores at 90 days post-stroke were not statistically worse than those in comparative historical studies, and at least one of these outcomes showed a numerical trend for improvement in the cooled patients. It should be emphasized that this analysis was not conducted to prove the efficacy of the cooling treatment but rather to determine whether a pivotal trial of the treatment should be undertaken in the future.

Stopping criteria were to be administered by the DSMB if necessary. The DSMB was to evaluate interim results of the study for evidence of efficacy or adverse events. The stopping rules as outlined in the FDA-approved protocol were as follows:

• Overall study population
 o Cooled patients have statistically worsened neurological outcomes vs. historical controls (NIHSS or mRS @ 90 days)
 o Cooled patients have statistically improved neurological outcomes vs. historical controls (NIHSS or mRS @ 90 days)
 o Cooled patients have a statistically higher rate of death vs. historical controls
 o Cooled patients have a statistically lower rate of death vs. historical controls
• Patients not receiving IVT or EVT

 o All stopping rules as defined for overall patient population

- Patients receiving IVT or EVT

 o All stopping rules as defined for overall patient population plus the following:
 o Cooled patients have statistically worsened rates of symptomatic intracranial hemorrhage (increase of 4 NIHSS points at 24 h) vs. historical controls
 o Cooled patients have statistically worsened rates of systemic hemorrhage causing hemodynamic instability

* Statistical significance to be defined as *p* < 0.05.

### 2.4 IRB/IDE approval

The SISCO study was conducted in compliance with the principles of the Declaration of Helsinki under an FDA–issued Investigational Device Exemption (IDE G130212) and Institutional Review Board (IRB) approvals from the Tulane Human Research Protection Office (16-978774), Geisinger Institutional Review Board (2019-0165), and Advarra IRB (CR00342456). All data were anonymized and de-identified. Written informed consent was obtained for all subjects

### 2.5 Description of participants

Demographic information, including patient age, sex, and race, were recorded for each study subject.

## 3 Results

Of patients presenting with strokes, 268 patients were screened for enrollment, of which 14 were enrolled from January 25, 2017, to June 2, 2023, from three comprehensive stroke centers (Tulane Medical Center, New Orleans, LA, Geisinger Medical Center, Danville, PA, and PRISMA Health/University of South Carolina, Columbia, SC). The reasons for exclusion of the remaining 254 patients are provided in Figure 1. Of the 14 patients who were enrolled, 13 were cooled, and one was excluded after consent due to a diagnosis of a stroke mimic. Two had protocol violations (late reperfusion interventions and cooling more than 8 h post-stroke for patients not meeting DAWN criteria), and one patient was lost to follow-up. Ten patients were included for the 90-day per protocol (PP) analyses, and 13 were included for intention-to-treat (ITT) data analysis. The ITT analysis demonstrated that the average NIHSS on admission was 18.7 (median was 16), and the average patient age was 62.7 (median was 65).

**Figure 2:**
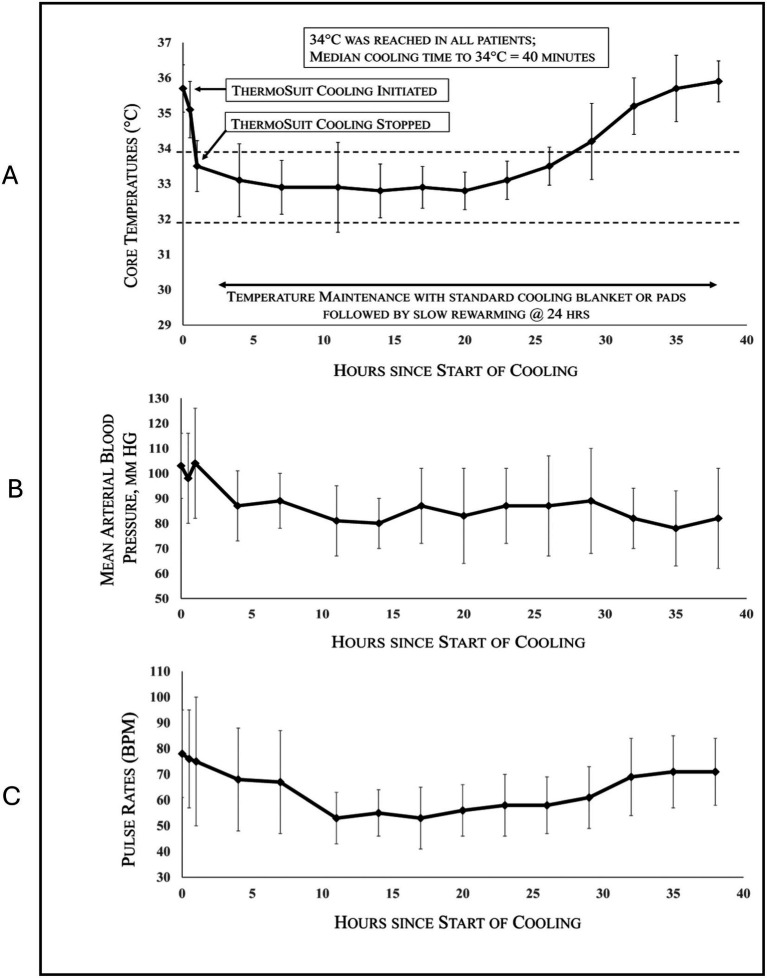
SISCO patient core temperatures **(A)**, mean arterial blood pressures **(B)**, and pulse rates **(C)** vs. hours after start of cooling (means ± SD) for all 13 cooled patients.

An overview of all patients (both PP and ITT) is provided in Table 2. Of the 10 PP patients with 90-day outcome data, anterior (seven), posterior (two), and PRES (posterior reversible encephalopathy syndrome)-related (one) stroke patients were enrolled. EVT was performed in seven of these patients, of which six also received IVT; two patients received IVT only, and one received neither. All patients reached the target temperature of 32–34 °C. Patient core temperatures, mean arterial blood pressures, and pulse rates for all 13 cooled patients are provided in Figure 2.

**Table 2:** Overview of SISCO patients.

Patient number and exclusions^a^	NIHSS	Age	Sex	Vascular territory	Interventions^a^		Time from EVT to cooling (h)	Time from EVT to 34 °C (h)	90-Day outcomes			
					IVT	EVT			EQ-5D	NIHSS	∆N	mRS
01 [1]	**…**	**…**	**…**	**…**	**…**	**…**	**…**	**…**	**…**	**…**	**…**	**…**
02	16	49	F	Bilateral PCA, PRES	**…**	**…**	**…**	**…**	0.20	1	−15	1
03	15	66	F	R vert/ BA	✓	**…**	**…**	**…**	0.86	1	−14	1
04	21	65	F	L MCA	✓	**…**	**…**	**…**	0.43	8	−13	3
05	21	70	M	L ICA	✓	✓	3.2	3.8	0.75	3	−18	2
06	13	77	F	BA	✓	✓	1.4	1.9	0.71	2	−11	3
07	14	59	F	R MCA	✓	✓	4.4	5.4	0.75	2	−12	2
08	16	70	F	R ICA	**…**	✓	2.1	2.9	0.83	2	−14	1
09	13	60	M	R ICA	✓	✓	3.5	3.9	N/A	N/A	N/A	N/A
10	28	65	F	L MCA	✓	✓	1.4	2.1	0.77	2	−26	3
11	12	59	F	R MCA	✓	✓	2.0	2.6	N/A	9	−3	4
12 [2]	32	59	M	BA	**…**	✓	4.0	4.6	0	42	+10	6
13 [3]	21	47	F	L MCA	**…**	✓	1.5	2.3	N/A	N/A	N/A	N/A
14	21	69	F	R ICA	✓	✓	3.9	4.3	N/A	N/A	N/A	3
Overall (per protocol)	17.3 Mean	64.3 Mean	82% Female	73% anterior, 27% posterior	82% w IVT	73% w EVT	2.6 h Mean	3.3 h Mean	0.75 Med	67% ≤2	−14 Mean	50% ≤2 90% ≤3
Overall (intention to treat)	18.7 Mean	62.7 Mean	77% Female	77% anterior, 23% posterior	69% w IVT	77% w EVT	2.7 h Mean	3.4 h Mean	0.75 Med	55% ≤2	−12 Mean	45% ≤2 82% ≤3

a Study exclusions.

[1] Stroke mimic.

[2] Time from stroke onset to reperfusion (8 h) and cooling (12.5 h) exceeded protocol maximums.

[3] Time from stroke onset to reperfusion (18 h) and cooling (19.5 h) exceeded protocol maximums.

SISCO, Helping Stroke Patients with ThermoSuit Cooling; NIHSS, National Institutes of Health Stroke Scale; IVT, Intravenous thrombolysis; ∆N, change in NIHSS from hospital admission to 90 days post-stroke; EVT, endovascular thrombectomy; mRS, modified Rankin score; PCA, posterior cerebral artery; MCA, middle cerebral artery; BA, Basilar artery; PRES, posterior reversible encephalopathy syndrome; EQ-5D, European Quality of Life Five Dimension to score.

The full mRS scale has values ranging from 0 (no symptoms) to 6 (dead); mRS ≤ 2 means disability is insignificant or slight; mRS ≤ 3 includes patients with mRS ≤ 2 plus those with moderate disability (requires some help, but able to walk unassisted).

### 3.1 Study endpoints

#### 3.1.1 Study endpoint 1—feasibility of cooling

**Figure 3:**
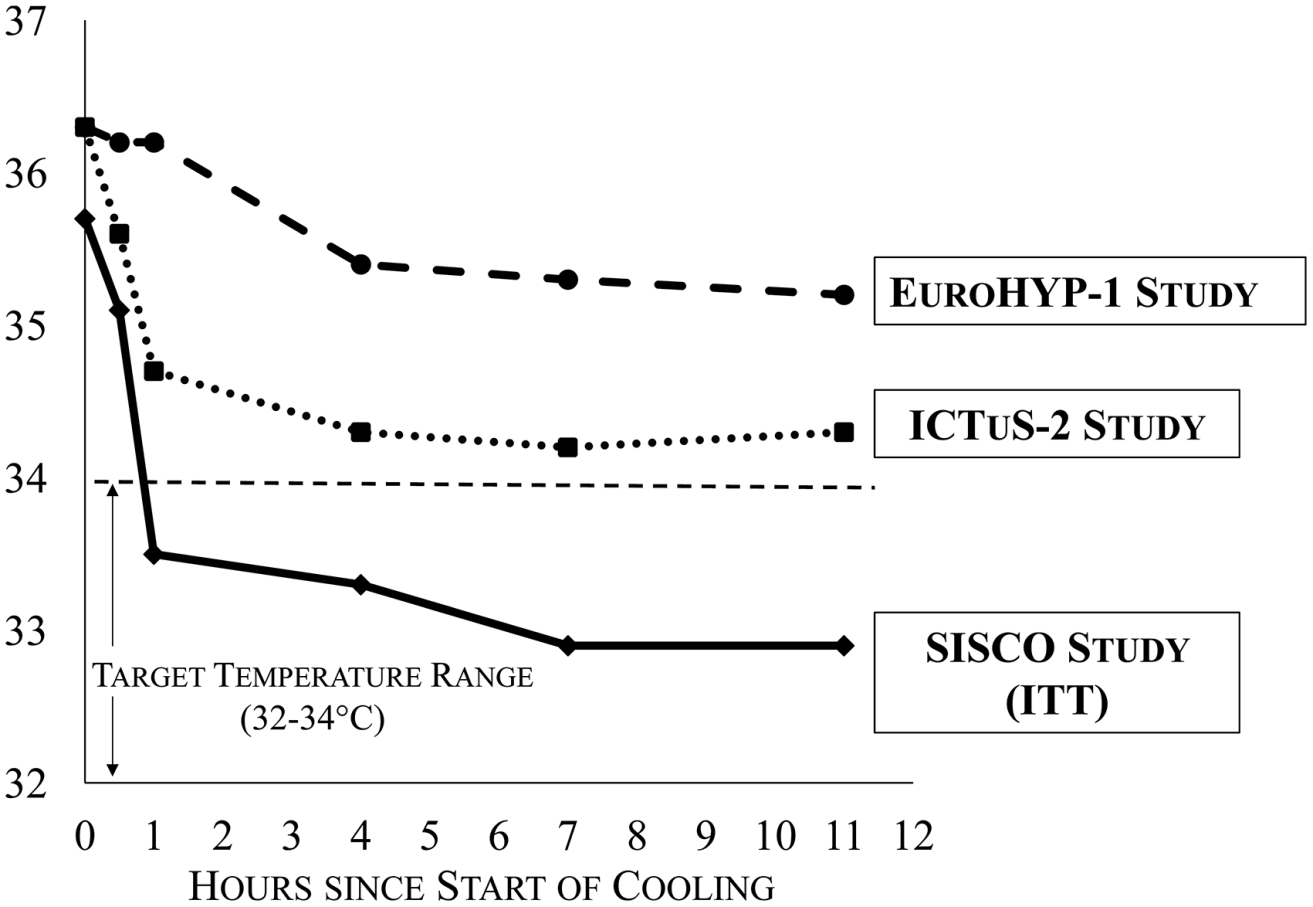
Mean core temperatures vs. time (SISCO vs. ICTuS-2 and EuroHYP-1 studies).

69% of subjects reached 34 °C within 1 h, and 93% of subjects reached 34 °C within 2 h. These results satisfied the pre-determined success criteria. The cooling time to 34 °C for ITT patients (mean ± SD) was 54 ± 34 min, and the median was 40 min. 100% of subjects reached 34 °C within 2.3 h. The cooling speeds achieved with the ThermoSuit device were markedly faster than those attained in the previous ICTuS 2 and EuroHYPE-1 studies (see Figure 3).

#### 3.1.2 Study endpoint 2—safety of the cooling treatment

The following adverse events were observed for the ITT (intention to treat) patients: bradycardia (3), malignant cerebral edema (1), progressive stroke (worsening of neurological function within 48 to 72 h after the onset of ischemic stroke, accompanied by an increase of NIHSS of 2 or more) (1), pneumonia (2), seizure (1), right upper extremity deep venous thrombosis (1), moderate acute pancreatitis (1), severe neurological deficits (indicated by an NIHSS of over 21 and acute symptoms such as paralysis, dysphagia, and unresponsiveness) (1), MRSA pneumonia (1), and mRS ≥ 4 (2). There were no deaths among the per-protocol patients, but one among the intention-to-treat patients.

#### 3.1.3 Study endpoint 3—neurological recoveries compared to comparative clinical trials

Seven of the ten PP SISCO patients for whom 90-day outcomes were available had anterior large vessel occlusions. The HERMES study (17) comprised 1,287 patients from five studies with anterior large vessel occlusions (half of which were treated with EVT).

Two PP SISCO patients had occlusions of the posterior circulation (vertebral and/or basilar arteries). Francalanza et al. (18) studied 27 patients treated for basilar strokes. One of the PP SISCO patients was enrolled and cooled after an initial observation of hypodensities in the posterior circulation. Later analysis indicated the likelihood of PRES in this patient, but a small permanent infarct was evident as well. Ismail et al. (19) reported on the results of 15 PRES patients. The patient ages and NIHSS scores at admission of the studies by the HERMES collaboration, Francalanza, and Ismail were not statistically different from those of the SISCO study (Table 3).

**Table 3:** Ischemic etiologies, ages, baseline NIHSS scores, and 90-day outcomes of ITT SISCO study subjects vs. those of comparative studies.

Baseline variables (below)	SISCO study	Comparative studies^a^			
	(11 TSS-cooled ITT subjects)	HERMES study (633 EVT subjects)	Francalanza study (27 subjects)	Ismail study (14 subjects)	Weighted averages of comparative studies^a^
Fraction of subjects with anterior strokes	7/11 (64%)	633/633 (100%)	0/27 (0%)	0/14 (0%)	64%
Fraction of patients with posterior strokes	3/11 (27%)	0/633 (0%)	27/27 (100%)	0/14 (0%)	27%
Fraction of subjects with PRES	1/11 (9%)	0/633 (0%)	0/27 (0%)	14/14 (100%)	9%
Patient age (median, IQR)	65 (59–69)	68 (57–77)	66 (59–73)	53 (35–62)	64 (58–67) *p* = 0.995 vs. SISCO (NS)
NIHSS on admission (median, IQR)	16 (14–21)	17 (14–20)	15 (11–19)	10	16 (15-16) *p* = 0.426 vs. SISCO (NS)
Fraction of subjects with mRS ≤ 2 @ 90 days	5/11 (45%)	291/633 (46.0%)	7/27 (25.9%)	6/14 (43%)	40%
Fraction of subjects with mRS ≤ 3 @ 90 days	9/11 (82%)	398/633 (62.9%)	11/27 (40.7%)	9/14 (64%)	57%^b^
Fraction of subjects who died	1/12 (8%)	97/634 (15.3%)	8/27 (29.6%)	2/14 (14%)	19%

a Published data from 674 subjects enrolled in HERMES Study (17), Francalanza Study (18), and Ismail study (19), weighted to match the intention to treat SISCO subjects (64% anterior, 27% posterior, and 9% PRES-related strokes).

b Sample calculation: Weighted average fraction of patients with mRS ≤ 3 from comparable studies for comparison with ITT SISCO subjects: (64%×62.9%)+(27%×40.7%)+(9%×64%)=57% .

ITT, intention to treat; mRS, modified Rankin scale; NIHSS, National Institutes of Health Stroke Scale; NS, not statistically significant.

The PP SISCO patient outcomes of mRS at 90 days and mortality were compared to those of the above studies by proportioning the published cases to reflect 70% with anterior strokes treated with EVT, 20% with posterior strokes, and 10% with PRES (as in the SISCO study). The TSS-cooled patients showed trends for higher rates of good and acceptable outcomes and a lower rate of mortality versus the subjects from comparable studies (Table 4). The strongest trend was a 55% increase in the rate of acceptable outcome (mRS ≤ 3) at 90 days in PP SISCO patients (RR = 1.55). In a similar comparison, 82% of ITT SISCO patients had acceptable outcomes, representing a 44% improvement compared to patients in comparative clinical trials (RR = 1.44).

**Table 4:** 90-Day outcomes of SISCO subjects compared to those of comparative studies.

Outcome categories	Outcomes of per protocol SISCO subjects	Weighted averages of outcomes of comparative clinical trials	Relative risks
mRS ≤ 2 @ 90 days	50% (5/10)	42%^a^	RR = 1.19
mRS ≤ 3 @ 90 days	90% (9/10)	58%^a^	RR = 1.55
Mortality	0% (0/10)	18%^a^	RR = N/A

Outcome categories	Outcomes of ITT SISCO subjects	Weighted averages of outcomes of comparative clinical trials	Relative risks
mRS ≤ 2 @ 90 days	45% (5/11)	40%^b^	RR = 1.14
mRS ≤ 3 @ 90 days	82% (9/11)	57%^b^	RR = 1.44
Mortality	8% (1/12)	19%^b^	RR = 0.44

a Published data from 674 subjects enrolled in HERMES study (17), Francalanza study (18), and Ismail study (19), weighted to match the per protocol SISCO subjects (70% anterior, 20% posterior, and 10% PRES-related strokes).

b Published data from 674 subjects enrolled in HERMES study (17), Francalanza study (18), and Ismail study (19), weighted to match the intention to treat SISCO subjects (64% anterior, 27% posterior, and 9% PRES-related strokes).

ITT, intention to treat; mRS, modified Rankin scale; RR, relative risk.

The PP SISCO patients included eight for whom quality of life results (EQ-5D) were available 90 days post-stroke and nine for whom 90-day NIHSS results were available. None of the studies by the HERMES collaboration, Ismail or Francalanza report these outcomes. The MR CLEAN study (20) was identified as having 90-day EQ-5D data that could be compared to the SISCO results, and the ESCAPE study (21) had 90-day NIHSS results. Comparative analyses showed trends favoring cooled patients for both outcomes; EQ-5D at 90 days were a median of 0.75 (IQR 0.71–0.77) for SISCO patients, and 0.69 (IQR 0.33–0.85) for comparable patients from the MR CLEAN study (*p* = 0.003). The second comparative analysis found that 67% of SISCO study patients had NIHSS scores of 0–2 at 90 days compared to 52% of the ESCAPE study patients (RR = 1.29, 95% CI = 0.794 to 2.101). This difference did not attain statistical significance (*p* = 0.500). NIHSS scores in PP SISCO patients improved by an average of 14 points from hospital admission to 90 days post-stroke, which was the same as ESCAPE study patients. Collectively, the above results confirmed that success criterion #3 was met.

## 4 Discussion

### 4.1 Speed, timing, and depth of cooling

The feasibility of rapidly cooling ischemic stroke patients with the ThermoSuit System to 32 to 34 °C was demonstrated. Compared to patients in ICTuS 2 and EuroHYP-1, patients in the SISCO study were cooled substantially faster and to a greater depth (Figure 3). These findings are important to consider, given that the ICTuS 2 (9) and EuroHYP-1 (10) studies reached 34 °C in very few patients and failed to detect beneficial outcomes associated with cooling. Notably, the earliest SISCO protocol required that the TSS purge temperature be set at 34.5 °C, halting the induction cooling prior to reaching the target temperature. The only patient requiring more than 2 h to reach the target temperature was under these conditions. This prompted the investigators to lower the purge temperature to 34.0 and 33.5 °C in later cases, which consistently produced rapid cooling to the target range. Consequently, the overall trial results may underestimate the overall TSS cooling speed.

**Figure 4:**
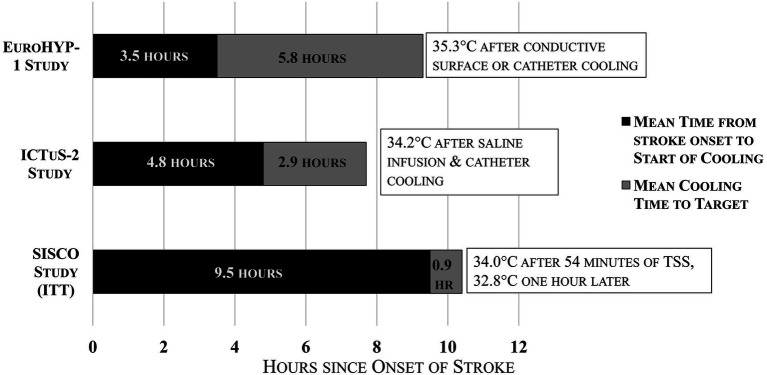
Times from stroke onset to start of cooling and time to target temperature for EuroHYP-1, ICTuS-2, and SISCO studies.

Despite rapid induction cooling demonstrated with TSS, the average time from stroke onset to 34 °C in the SISCO Study, on an intention-to-treat basis, was 9.5 h. This delay was partially due to the inclusion of DAWN criteria for extended-window thrombectomy in addition to the requirement that cooling be initiated post-reperfusion only. One patient was cooled 27.7 h after stroke onset (patient no. 8), and two others were cooled late and categorized as protocol violations (patients nos. 12 and 13). Figure 4 shows a comparison of average times to cooling for the EuroHYPE-1, ICTuS-2 and SISCO studies. The late cooling induction may have been counterbalanced by the faster and deeper temperature drop provided by the TSS treatment. Prior research has shown that ischemic neurons can survive for hours using anerobic metabolism (22). However, reperfusion injury leads to cell death that progresses rapidly within the first 4 h after reperfusion (23–25).

Data provided in Table 2 shows that among the eight patients treated with EVT, the mean time from intervention completion to the start of cooling in the SISCO study was 2.7 h. These patients required an additional mean time of 0.7 h to be cooled to 34 °C by the TSS. Thus, the average time from EVT completion to 34 °C was 3.4 h. The post-EVT-to-cooling delay was partly related to study-related activities, such as gathering appropriate study personnel and obtaining informed consent as required in this FDA-regulated study. Multiple animal models of therapeutic hypothermia in stroke demonstrated that cooling delay substantially diminishes the treatment effect, and benefit may be lost entirely 6 h after reperfusion (5, 23).

In future clinical applications of the TSS cooling therapy, reducing the time between EVT and the start of cooling to less than 60 min would be strongly recommended to enable TH to be reached within 2 h after reperfusion. Alternatively, animal data suggests that pre-reperfusion cooling may offer even further reductions in infarct size compared to post-reperfusion cooling (24). This pilot trial has demonstrated the speed and safety of TSS cooling that could allow for future efficacy trial designs to consider pre-reperfusion cooling among patients who receive EVT, especially in those patients with delays to EVT (e.g., long transport times) or during reperfusion with IVT. A future trial may also consider infarct size as a secondary outcome, using volumetric analysis abstracted from CT or MRI studies. Overall, we hypothesize that earlier and faster cooling approaches would result in smaller infarct volumes and better outcomes.

### 4.2 Safety and outcomes

Adverse events in the ITT SISCO study patients were compared to those of the MR CLEAN study, in which EVT was provided but cooling was not (20). The results of this comparison are shown in Table 5. Bradycardia was observed in three SISCO subjects and was asymptomatic, but it resolved upon rewarming in all cases.

**Table 5:** Comparisons of adverse events of SISCO (intention to treat) and MR clean study.

Patient characteristics	SISCO study patients (*N* = 13) with cooling^a^	MR CLEAN study patients (*N* = 233) without cooling^b^	Differences between SISCO and MR clean study results	*P* values
NIHSS on Admission (median, IQR)	16 (14–21)	17 (14–21)	Difference between the medians of the two groups: 1	*p* = 0.076
Patient Age (median, IQR)	65 (59–69)	65.8 (54.5–76.0)	Difference between the medians of the two groups: 0.8	*p* = 0.363
Malignant cerebral edema	1/13 (7.7%)	14/233 (6.0%)	RR = 1.28 (95% CI = 0.182 to 9.001)	*p* = 0.568
Progressive stroke	1/13 (7.7%)	46/233 (20%)	RR = 0.39 (95% CI = 0.058 to 2.607)	*p* = 0.472
Pneumonia	3/13 (23%)	25/233 (11%)	RR = 2.15 (95% CI = 0.746 to 6.204)	*p* = 0.173
Serious disability, coma, or death at 90 days (mRS ≥ 4)	2/11 (18%)	114/233 (49%)	RR = 0.37 (95% CI = 0.105 to 1.311)	*p* = 0.063*

a 78% anterior stroke patients with EVT, 15% posterior stroke patients, 7% PRES patients, all cooled with ThermoSuit; 13 patients were available for in-hospital analyses, but only 11 were available for 90-day analyses.

b Anterior stroke patients receiving EVT but without cooling.

*Statistically significant using one-tailed *p*-value (0.043) but not significant using two-tailed *p*-value (0.063).

CI, confidence interval; NIHSS, National Institutes of Health Stroke Scale; RR, relative risk; PRES, posterior reversible encephalopathy syndrome.

The incidence of pneumonia in the MR CLEAN study patients who received EVT was 11%, which was not statistically different from the 23% incidence observed in the SISCO study. For comparison, the rates of pneumonia in the cooled patients of the ICTuS 2 and EuroHyp1 studies were 19 and 18%, respectively. The 18% incidence of severe disability, coma, or death (mRS ≥ 4) at 90 days in ITT patients was well below the 49% incidence in other clinical trials who received EVT, but this difference did not attain statistical significance. In a similar analysis of the PP SISCO patients, the incidence of mRS ≥ 4 was 10%, yielding a significant benefit [RR = 0.20 (95% CI = 0.04 to 0.98), *p* = 0.0021].

The impact of rapid cooling on neurological outcomes was assessed by comparing the SISCO study results to those of published studies of comparable non-cooled stroke patients. The above favorable trends are suggestive that an adequately powered prospectively randomized study could show significantly improved outcomes associated with the TSS cooling treatment in acute ischemic stroke patients, mainly if mRS ≤ 3 or EQ-5D is selected as the primary outcome. A sample size estimate for a possible future pivotal study was calculated based on comparison of the percentage of acceptable outcomes for intention to treat SISCO patients (82%) vs. the percentage of acceptable outcomes from comparable studies (57%). The calculation was made using a test for two proportions, assuming 90% power at *α* = 0.05. This analysis suggested that a significant benefit for the cooling treatment could be proven with a pivotal trial enrolling approximately 114 patients. This estimate corresponds to an RR of 1.44. Additional study size estimates with more conservative assumptions should be considered prior to initiating a definitive, prospectively randomized trial. For example, if the RR is downgraded to 1.41, the required sample size would increase to 150. If 6% of study patients are lost to attrition, the targeted sample size would need to be 160. Additional analyses, utilizing a Bayesian approach and Monte Carlo simulations, are in progress and will be completed prior to initiation of the definitive trial.

Many lessons learned from individual patients allowed for protocol modification in this small pilot trial. Most patients were enrolled at a single center with two investigators (11/14 patients, 78.6%).

The earliest protocols allowed for intervention without intubation, and one patient underwent successful cooling without it. Sedation was administered with dexmedetomidine and low-dose midazolam. Shivering was minimal and no immediate complications were noted. Despite a good outcome, the investigators selected future patients who had a non-study-related indication for intubation, such as airway protection due to neurological condition or for thrombectomy. No patients were intubated solely for study purposes. The investigators recommend that a future trial require intubation for all cooled patients.

One individual had early cerebral edema while at the target temperature. The treatment team decided that surgery was emergent, and rapid rewarming would likely increase cerebral edema, resulting in herniation. Based on this, the patient underwent decompressive hemicraniectomy while the temperature was maintained from 32 to 34 °C (26). There were no bleeding complications, and the patient had an outcome better than expected for those with malignant MCA syndrome: modified Rankin score of 2 and living with family. The literature on bleeding risk and hypothermia is based primarily on trauma and major surgical interventional settings in which additional confounding effects may affect coagulation and bleeding (27). Furthermore, a subset of patients enrolled in a randomized control trial of mild therapeutic hypothermia in sepsis (33–34 °C for 24 h) were evaluated with thrombo-elastography (TEG) and found that therapeutic hypothermia improved sepsis-induced coagulopathy when compared to control patients at euthermia; this improvement lasted beyond the intervention phase (28). Similarly, a meta-analysis of 7,258 patients concluded that therapeutic hypothermia did not increase the risk of hemorrhage (29). These findings question the long-standing assumption that bleeding risk is increased by a lower body temperature.

The single mortality was a man, 59 years old, who suffered a mid-basilar occlusion and had significant delays in transport time from the sending facility. He received EVT with TICI 2b flow at 8 h from stroke onset and had cooling initiation at 12.5 h. Despite the interventions, he had a progressive neurological decline and succumbed to a massive posterior circulation stroke. This case highlights the need for future studies to consider a “cool and ship model” whereby cooling is initiated at outlying hospitals, potentially improving neuronal tolerance to prolonged ischemia and reperfusion.

### 4.3 Shivering

Studies of cooling patients without paralytics report a nearly 100% incidence of shivering, typically between 33.5 and 35.5 °C (9, 10, 30). The EuroHYP-1 study investigators attempted to suppress shivering using meperidine and buspirone, but this was effective in only 31% of patients; the majority of the remaining patients could not maintain temperatures below 35.0 °C for more than 6 h. Similarly, the ICTuS 2/3 protocol, which included buspirone, meperidine, and skin warming, allowed switching to a higher target temperature if shivering could not be controlled. Consequently, most patients failed to reach 34 °C.

In contrast, the SISCO Study swiftly achieved target temperatures below 34 °C in all patients using the TSS without meperidine and only three patients required paralytics. While 12/13 (92%) of SISCO patients experienced some shivering, patients shivered only 5.4% of the study time, 9.2% of the time within the range of 33.1 to 35.5 °C and just 1.3% of the time below 33.1 °C (see Table 6). This represents an overall reduction in shivering time of approximately 90% compared to the slower cooling methods that used meperidine, buspirone, and skin warming (9, 10). The median duration of shivering during TSS treatment was 0 h, IQR 0 to 30 min (mean = 7.5 min). The TSS likely suppressed shivering due to cold water contact and rapid cooling (31). Cooling the skin below 8 °C reduces cutaneous cold-firing receptor activity (32), one of the primary drivers of shivering.

**Table 6:** Overview of shivering in cooled SISCO patients during therapeutic hypothermia^a^.

Patient core temperature range (first 25 h)	Total hours in range for all cooled patients	Total hours of shivering in range for all cooled patients	Percentage of time shivering occurred in range
> 35.5 °C	7	1.2	17.1%
35.5 °C–33.1 °C	158	14.6	9.2%
≤33.0 °C	163	2.2	1.3%
All temperatures	328	18.0	5.4%

a Includes all 13 ischemic stroke patients cooled with ThermoSuit System.

Median duration of shivering during TSS cooling induction (liquid convection) = 0 h (mean = 0.12 h).

Median duration of shivering during maintenance TH (conductive cooling/warming) = 0.17 h (mean = 1.25 h).

Propofol and fentanyl were used in all subjects for sedation and patient comfort during cooling induction. Propofol has the additional benefits of facilitating cooling and reducing shivering (33). A recent survey of clinical centers performing EVT found that propofol was the most frequently used anesthetic induction agent (34). Other drugs that were used with less frequency in the SISCO study included dexmedetomidine, midazolam, lorazepam, and magnesium sulfate. Buspirone was used in half of the cases to prevent shivering during temperature maintenance. While meperidine is commonly used to lower the shivering threshold (35), it was avoided in this study as it has been previously associated with increased cerebrospinal fluid pressure and the incidence of respiratory problems (36).

### 4.4 Limitations

The SISCO Study was a small, uncontrolled, unblinded pilot study to assess the safety and feasibility of cooling ischemic stroke patients with the TSS. The results have reached pre-specified endpoints for this feasibility study with planned future randomized trials. Life Recovery Systems oversaw the study, which included design, planning, and analysis. Bias was minimized with the addition of other investigators. Patients were selected for the study based on the availability of investigators to implement treatment and record findings. Enrollment was slow and halted during the COVID-19 pandemic as resources were limited for clinical trials.

Study enrollment was slow. Besides the problems caused by the COVID-19 pandemic, the informed consent process was burdensome. Two of the three study hospitals required in-person consent by a legally authorized representative—a nearly impossible task. The third hospital had an abbreviated remote consent process; this should be considered for use in future studies. In addition, the study inclusion criteria were very restrictive. These were largely based on safety-related concerns of both the FDA and investigators regarding the untested treatment. Based on the favorable experience gained in this study, it should be possible to loosen some of these restrictions, such as those directed at minimizing conditions conducive to bleeding. As shown on Figure 1, 44 patients were excluded from the study due to their receiving thrombin or factor Xa inhibitors; however, no significant bleeding was observed in any patient in this study that was associated with drugs or the cooling treatment. We believe that this restriction should be removed in future applications of this noninvasive treatment. More significantly, 64 patients were excluded due to study staff being unavailable. It is recommended that study staffing be significantly increased in the pivotal trial so that more patients can be enrolled.

### 4.5 Study termination

Based on the results for cooling speed, outcomes, and safety, all predetermined criteria for the success of the SISCO study were satisfied. The trial was terminated early after enrolling 14 of the initially planned 30 subjects due to satisfying predetermined criteria for trial success. The FDA recommended moving forward with a pivotal trial to thoroughly investigate the use of the TSS for the treatment of ischemic stroke patients.

## 5 Conclusion

The TSS is a feasible, and effective tool for rapidly inducing TH in ischemic stroke patients. This noninvasive cooling technique allows clinicians to swiftly cool patients below 34 °C with relatively little shivering and no apparent safety issues. Trends toward improved clinical outcomes were observed compared to matched previously published stroke trials, but this feasibility study was not designed or powered to draw beneficial conclusions. Further studies are warranted to prove whether this rapid and safe cooling method is neuroprotective in stroke patients.

## Data Availability

The raw data supporting the conclusions of this article will be made available by the authors, without undue reservation.
